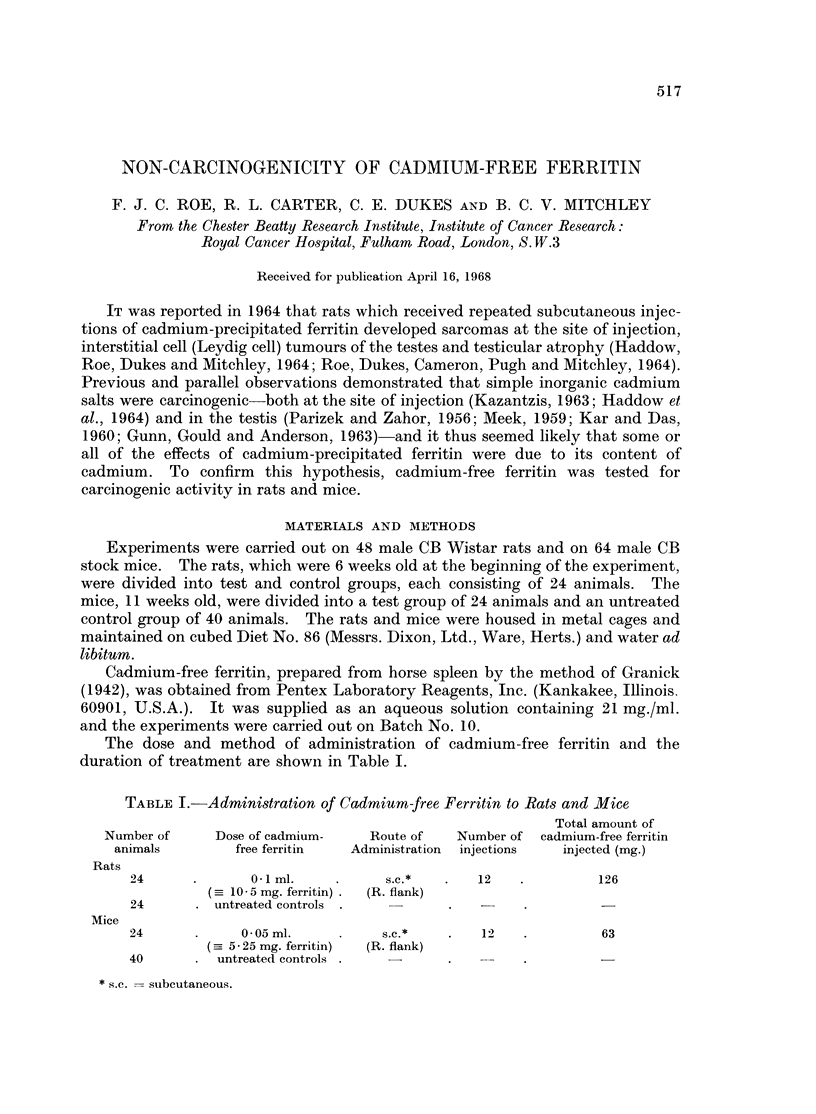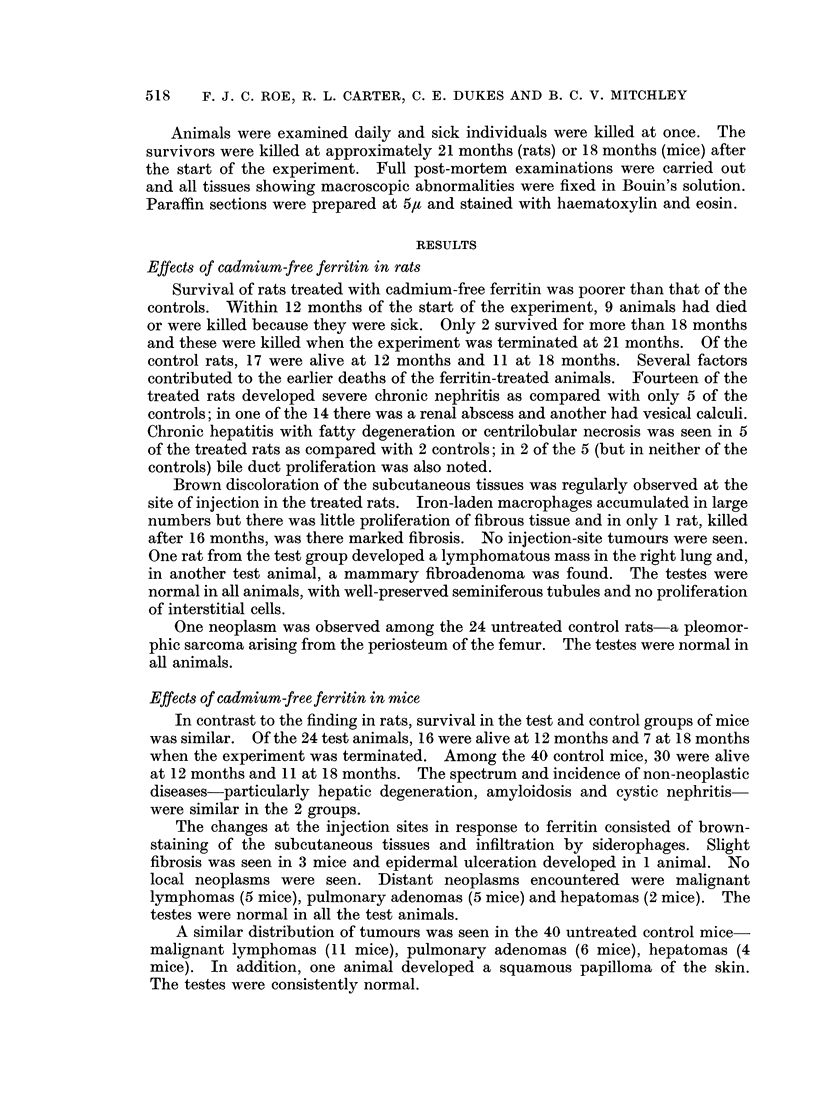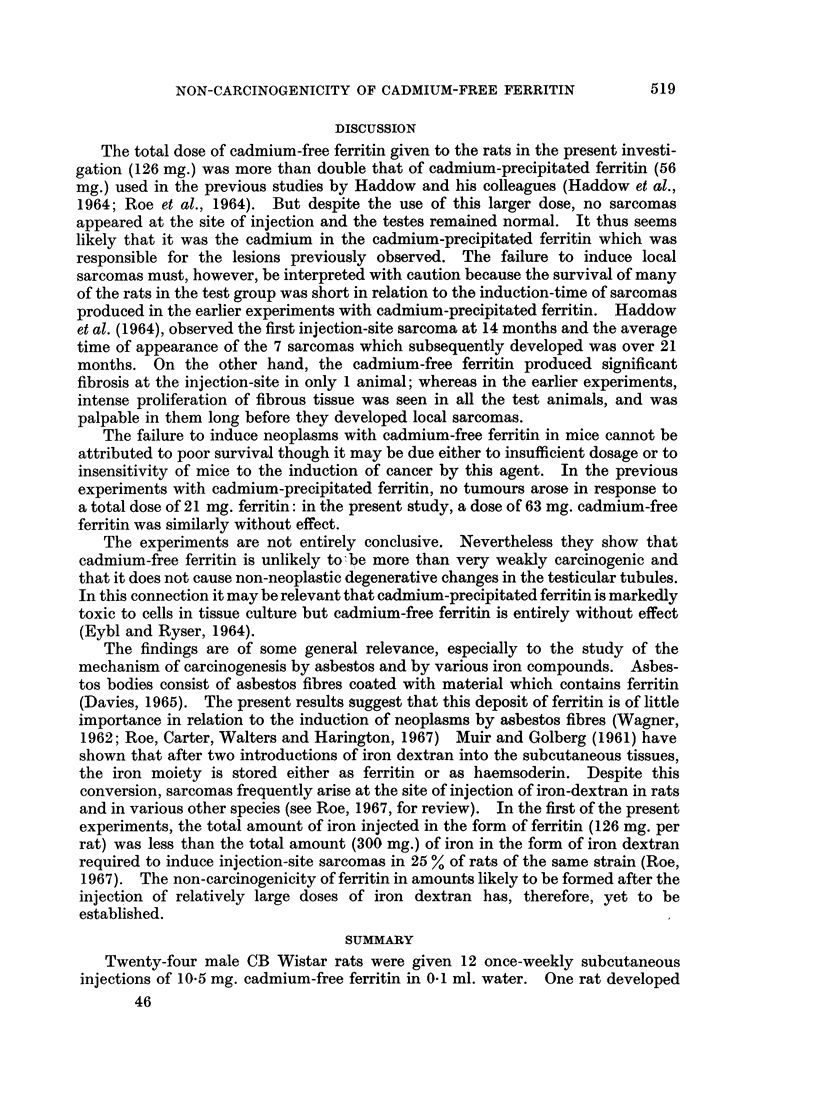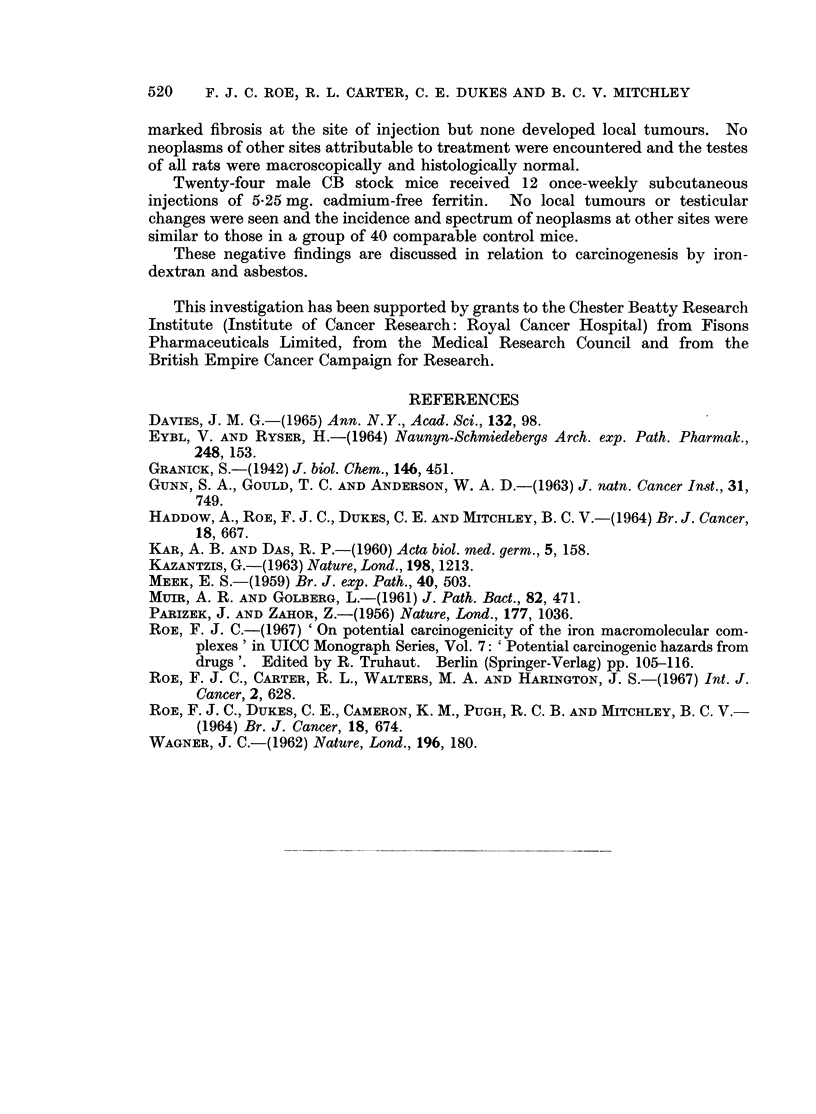# Non-carcinogenicity of cadmium-free ferritin.

**DOI:** 10.1038/bjc.1968.61

**Published:** 1968-09

**Authors:** F. J. Roe, R. L. Carter, C. E. Dukes, B. C. Mitchley


### NON-CARCINOGENICITY OF CADMIUM-FREE FERRITIN

F. J. C. ROE, R. L. CARTER, C. E. DUKES AND B. C. V. MITCHLEY
*From the Chester Beatty Research Institute, Institute of Cancer Research: Royal Cancer Hospital, Fulham Road, London, S.W.3*

Received for publication April 16, 1968

IT was reported in 1964 that rats which received repeated subcutaneous injections of cadmium-precipitated ferritin developed sarcomas at the site of injection, interstitial cell (Leydig cell) tumours of the testes and testicular atrophy (Haddow, Roe, Dukes and Mitchley, 1964; Roe, Dukes, Cameron, Pugh and Mitchley, 1964). Previous and parallel observations demonstrated that simple inorganic cadmium salts were carcinogenic—both at the site of injection (Kazantzis, 1963; Haddow *et al.*, 1964) and in the testis (Parizek and Zahor, 1956; Meek, 1959; Kar and Das, 1960; Gunn, Gould and Anderson, 1963)—and it thus seemed likely that some or all of the effects of cadmium-precipitated ferritin were due to its content of cadmium. To confirm this hypothesis, cadmium-free ferritin was tested for carcinogenic activity in rats and mice.

### MATERIALS AND METHODS

Experiments were carried out on 48 male CB Wistar rats and on 64 male CB stock mice. The rats, which were 6 weeks old at the beginning of the experiment, were divided into test and control groups, each consisting of 24 animals. The mice, 11 weeks old, were divided into a test group of 24 animals and an untreated control group of 40 animals. The rats and mice were housed in metal cages and maintained on cubed Diet No. 86 (Messrs. Dixon, Ltd., Ware, Herts.) and water *ad libitum.*

Cadmium-free ferritin, prepared from horse spleen by the method of Granick (1942), was obtained from Pentex Laboratory Reagents, Inc. (Kankakee, Illinois, 60901, U.S.A.). It was supplied as an aqueous solution containing 21 mg./ml. and the experiments were carried out on Batch No. 10.

The dose and method of administration of cadmium-free ferritin and the duration of treatment are shown in Table I.

TABLE I.—*Administration of Cadmium-free Ferritin to Rats and Mice*

| Number of animals | Dose of cadmium-free ferritin | Route of Administration | Number of injections | Total amount of cadmium-free ferritin injected (mg.) |
|---|---|---|---|---|
| Rats | | | | |
| 24 | 0·1 ml. (≡ 10·5 mg. ferritin) | s.c.* (R. flank) | 12 | 126 |
| 24 | untreated controls | — | — | — |
| Mice | | | | |
| 24 | 0·05 ml. (≡ 5·25 mg. ferritin) | s.c.* (R. flank) | 12 | 63 |
| 40 | untreated controls | — | — | — |

* s.c. = subcutaneous.

Animals were examined daily and sick individuals were killed at once. The survivors were killed at approximately 21 months (rats) or 18 months (mice) after the start of the experiment. Full post-mortem examinations were carried out and all tissues showing macroscopic abnormalities were fixed in Bouin's solution. Paraffin sections were prepared at $5\mu$ and stained with haematoxylin and eosin.

### RESULTS

### *Effects of cadmium-free ferritin in rats*

Survival of rats treated with cadmium-free ferritin was poorer than that of the controls. Within 12 months of the start of the experiment, 9 animals had died or were killed because they were sick. Only 2 survived for more than 18 months and these were killed when the experiment was terminated at 21 months. Of the control rats, 17 were alive at 12 months and 11 at 18 months. Several factors contributed to the earlier deaths of the ferritin-treated animals. Fourteen of the treated rats developed severe chronic nephritis as compared with only 5 of the controls; in one of the 14 there was a renal abscess and another had vesical calculi. Chronic hepatitis with fatty degeneration or centrilobular necrosis was seen in 5 of the treated rats as compared with 2 controls; in 2 of the 5 (but in neither of the controls) bile duct proliferation was also noted.

Brown discoloration of the subcutaneous tissues was regularly observed at the site of injection in the treated rats. Iron-laden macrophages accumulated in large numbers but there was little proliferation of fibrous tissue and in only 1 rat, killed after 16 months, was there marked fibrosis. No injection-site tumours were seen. One rat from the test group developed a lymphomatous mass in the right lung and, in another test animal, a mammary fibroadenoma was found. The testes were normal in all animals, with well-preserved seminiferous tubules and no proliferation of interstitial cells.

One neoplasm was observed among the 24 untreated control rats—a pleomorphic sarcoma arising from the periosteum of the femur. The testes were normal in all animals.

### *Effects of cadmium-free ferritin in mice*

In contrast to the finding in rats, survival in the test and control groups of mice was similar. Of the 24 test animals, 16 were alive at 12 months and 7 at 18 months when the experiment was terminated. Among the 40 control mice, 30 were alive at 12 months and 11 at 18 months. The spectrum and incidence of non-neoplastic diseases—particularly hepatic degeneration, amyloidosis and cystic nephritis—were similar in the 2 groups.

The changes at the injection sites in response to ferritin consisted of brown-staining of the subcutaneous tissues and infiltration by siderophages. Slight fibrosis was seen in 3 mice and epidermal ulceration developed in 1 animal. No local neoplasms were seen. Distant neoplasms encountered were malignant lymphomas (5 mice), pulmonary adenomas (5 mice) and hepatomas (2 mice). The testes were normal in all the test animals.

A similar distribution of tumours was seen in the 40 untreated control mice—malignant lymphomas (11 mice), pulmonary adenomas (6 mice), hepatomas (4 mice). In addition, one animal developed a squamous papilloma of the skin. The testes were consistently normal.

### DISCUSSION

The total dose of cadmium-free ferritin given to the rats in the present investigation (126 mg.) was more than double that of cadmium-precipitated ferritin (56 mg.) used in the previous studies by Haddow and his colleagues (Haddow *et al.*, 1964; Roe *et al.*, 1964). But despite the use of this larger dose, no sarcomas appeared at the site of injection and the testes remained normal. It thus seems likely that it was the cadmium in the cadmium-precipitated ferritin which was responsible for the lesions previously observed. The failure to induce local sarcomas must, however, be interpreted with caution because the survival of many of the rats in the test group was short in relation to the induction-time of sarcomas produced in the earlier experiments with cadmium-precipitated ferritin. Haddow *et al.* (1964), observed the first injection-site sarcoma at 14 months and the average time of appearance of the 7 sarcomas which subsequently developed was over 21 months. On the other hand, the cadmium-free ferritin produced significant fibrosis at the injection-site in only 1 animal; whereas in the earlier experiments, intense proliferation of fibrous tissue was seen in all the test animals, and was palpable in them long before they developed local sarcomas.

The failure to induce neoplasms with cadmium-free ferritin in mice cannot be attributed to poor survival though it may be due either to insufficient dosage or to insensitivity of mice to the induction of cancer by this agent. In the previous experiments with cadmium-precipitated ferritin, no tumours arose in response to a total dose of 21 mg. ferritin: in the present study, a dose of 63 mg. cadmium-free ferritin was similarly without effect.

The experiments are not entirely conclusive. Nevertheless they show that cadmium-free ferritin is unlikely to be more than very weakly carcinogenic and that it does not cause non-neoplastic degenerative changes in the testicular tubules. In this connection it may be relevant that cadmium-precipitated ferritin is markedly toxic to cells in tissue culture but cadmium-free ferritin is entirely without effect (Eybl and Ryser, 1964).

The findings are of some general relevance, especially to the study of the mechanism of carcinogenesis by asbestos and by various iron compounds. Asbestos bodies consist of asbestos fibres coated with material which contains ferritin (Davies, 1965). The present results suggest that this deposit of ferritin is of little importance in relation to the induction of neoplasms by asbestos fibres (Wagner, 1962; Roe, Carter, Walters and Harington, 1967) Muir and Golberg (1961) have shown that after two introductions of iron dextran into the subcutaneous tissues, the iron moiety is stored either as ferritin or as haemsoderin. Despite this conversion, sarcomas frequently arise at the site of injection of iron-dextran in rats and in various other species (see Roe, 1967, for review). In the first of the present experiments, the total amount of iron injected in the form of ferritin (126 mg. per rat) was less than the total amount (300 mg.) of iron in the form of iron dextran required to induce injection-site sarcomas in 25 % of rats of the same strain (Roe, 1967). The non-carcinogenicity of ferritin in amounts likely to be formed after the injection of relatively large doses of iron dextran has, therefore, yet to be established.

### SUMMARY

Twenty-four male CB Wistar rats were given 12 once-weekly subcutaneous injections of 10·5 mg. cadmium-free ferritin in 0·1 ml. water. One rat developed marked fibrosis at the site of injection but none developed local tumours. No neoplasms of other sites attributable to treatment were encountered and the testes of all rats were macroscopically and histologically normal.

Twenty-four male CB stock mice received 12 once-weekly subcutaneous injections of 5·25 mg. cadmium-free ferritin. No local tumours or testicular changes were seen and the incidence and spectrum of neoplasms at other sites were similar to those in a group of 40 comparable control mice.

These negative findings are discussed in relation to carcinogenesis by iron-dextran and asbestos.

This investigation has been supported by grants to the Chester Beatty Research Institute (Institute of Cancer Research: Royal Cancer Hospital) from Fisons Pharmaceuticals Limited, from the Medical Research Council and from the British Empire Cancer Campaign for Research.

### REFERENCES

DAVIES, J. M. G.—(1965) *Ann. N.Y., Acad. Sci.*, **132**, 98.

EYBL, V. AND RYSER, H.—(1964) *Naunyn-Schmiedebergs Arch. exp. Path. Pharmak.*, **248**, 153.

GRANICK, S.—(1942) *J. biol. Chem.*, **146**, 451.

GUNN, S. A., GOULD, T. C. AND ANDERSON, W. A. D.—(1963) *J. natn. Cancer Inst.*, **31**, 749.

HADDOW, A., ROE, F. J. C., DUKES, C. E. AND MITCHLEY, B. C. V.—(1964) *Br. J. Cancer*, **18**, 667.

KAR, A. B. AND DAS, R. P.—(1960) *Acta biol. med. germ.*, **5**, 158.

KAZANTZIS, G.—(1963) *Nature, Lond.*, **198**, 1213.

MEEK, E. S.—(1959) *Br. J. exp. Path.*, **40**, 503.

MUIR, A. R. AND GOLBERG, L.—(1961) *J. Path. Bact.*, **82**, 471.

PARIZEK, J. AND ZAHOR, Z.—(1956) *Nature, Lond.*, **177**, 1036.

ROE, F. J. C.—(1967) ' On potential carcinogenicity of the iron macromolecular complexes ' in UICC Monograph Series, Vol. 7: ' Potential carcinogenic hazards from drugs '. Edited by R. Truhaut. Berlin (Springer-Verlag) pp. 105–116.

ROE, F. J. C., CARTER, R. L., WALTERS, M. A. AND HARINGTON, J. S.—(1967) *Int. J. Cancer*, **2**, 628.

ROE, F. J. C., DUKES, C. E., CAMERON, K. M., PUGH, R. C. B. AND MITCHLEY, B. C. V.—(1964) *Br. J. Cancer*, **18**, 674.

WAGNER, J. C.—(1962) *Nature, Lond.*, **196**, 180.